# Impact of Rapid Thermal Annealing and Oxygen Concentration on Symmetry Bipolar Switching Characteristics of Tin Oxide-Based Memory Devices

**DOI:** 10.3390/mi16080956

**Published:** 2025-08-19

**Authors:** Kai-Huang Chen, Chien-Min Cheng, Ming-Cheng Kao, Hsin-Chin Chen, Yao-Chin Wang, Yu-Han Tsai

**Affiliations:** 1 Department of Electronic Engineering, Cheng Shiu University, Kaohsiung 83347, Taiwan; 0662@gcloud.csu.edu.tw (H.-C.C.); y.c.wang@ieee.org (Y.-C.W.); 2 Department of Electronic Engineering, Southern Taiwan University of Science and Technology, Tainan 71005, Taiwan; ma530221@stust.edu.tw; 3 Graduate Institute of Aeronautics, Department of Information and Communication Engineering, Chaoyang University of Technology, Taichung 413310, Taiwan; kmc@cyut.edu.tw

**Keywords:** symmetry bipolar switching, the tin oxide (SnO_2_), resistance random-access memory, electronic conduction mechanism, *I*–*V*

## Abstract

In this study, tin oxide (SnO_2_) resistive random-access memory (RRAM) thin films were fabricated using the thermal evaporation and radiofrequency and dc frequency sputtering techniques for metal–insulator–metal (MIM) structures. The fabrication process began with the deposition of a silicon dioxide (SiO_2_) layer onto a silicon (Si) substrate, followed by the deposition of a titanium nitride (TiN) layer to serve as the bottom electrode. Subsequently, the tin oxide (SnO_2_) layer was deposited as the resistive switching insulator. Two types of top electrodes were developed to investigate the influence of different oxygen concentrations on the bipolar switching, electrical characteristics, and performance of memory devices. An aluminum (Al) top electrode was deposited using thermal evaporation, while a platinum (Pt) top electrode was deposited via dc sputtering. As a result, two distinct metal–insulator–metal (MIM) memory RRAM device structures were formed, i.e., Al/SnO_2_/TiN/SiO_2_/Si and Pt/SnO_2_/TiN/SiO_2_/Si. In addition, the symmetry bipolar switching characteristics, electrical conduction mechanism, and oxygen concentration factor of the tin oxide-based memory devices using rapid thermal annealing and different top electrodes were determined and investigated by ohmic, space-charge-limit-current, Schottky, and Poole–Frenkel conduction equations in this study.

## 1. Introduction

Computer memory refers to devices that store electronic data signals through technologies such as semiconductors and magnetic media. In electronic circuits, data are stored in the binary format. The basic unit of each type of memory product may vary depending on the technology, but in general, memory devices are classified into volatile memory and non-volatile memory (NVM) devices based on their data retention capabilities relative to power supply. Traditional memory architectures have primarily relied on volatile memory, which includes Static Random-Access Memory (SRAM) and Dynamic Random-Access Memory (DRAM). These memory types offer high-speed access and virtually unlimited read/write endurance, making them fundamental to computing systems from the PC era to present-day applications. However, a major limitation of SRAM and DRAM is their dependence on continuous power; once the power supply is removed, the stored data are instantly lost. Moreover, even in the standby mode, a constant power supply is required to maintain data, resulting in leakage current and significant energy consumption. In contrast, with the rapid advancement of low-power electronic systems such as the Internet of Things (IoT), energy harvesting devices, and wireless sensor networks (WSNs), the industry is witnessing a shift toward non-volatile memory technologies as the next-generation mainstream memory solutions. These memory types retain data without power, offering critical advantages in energy efficiency and enabling persistent data storage in intermittently powered systems [1,2,3,4].

The data storage mechanism of non-volatile memory (NVM) relies on either the floating gate structure of specialized semiconductor devices or a resistance switching mechanism that differentiates between high and low resistance states to represent binary data (0 and 1). These mechanisms allow stored information to be retained even when power is removed during standby, eliminating energy consumption while preserving data integrity. However, as semiconductor devices continue to scale down in accordance with Moore’s Law, flash memory is increasingly facing physical limitations. One significant challenge arises as the tunneling oxide layer becomes thinner, leading to slower data operation speeds and reduced endurance of the memory cells. As a result, the development of novel and advanced non-volatile memory technologies has become a critical research focus. Emerging next-generation NVM technologies can be broadly categorized into four types: the Magnetoresistive RAM (MRAM), Phase Change Memory (PCM), Ferroelectric RAM (FeRAM), and Resistive RAM (RRAM) devices [1,2,3,4].

Among these, Resistive RAM (RRAM) has garnered the most attention as a key research direction due to its outstanding potential. After a comparative analysis of the characteristics of emerging memory technologies, resistive random-access memory (RRAM) demonstrates several notable advantages. Compared to Magnetoresistive RAM (MRAM), the RRAM exhibits lower power consumption, shorter write and read times, supports multi-level data storage, and features a smaller device footprint. Compared to Phase Change Memory (PCM), the RRAM offers faster erase times, greater endurance, and smaller feature sizes. Compared to Ferroelectric RAM (FeRAM), the RRAM provides faster write and read speeds, multi-bit storage capability, and more compact scaling potential. Based on these comparative advantages, RRAM stands out as the most promising among next-generation non-volatile memory technologies.

Especially in the advantages of resistive random-access memory, for beyond the widely recognized benefits of low fabrication cost, structural simplicity, high integration density, and low power consumption, resistive switching in resistive random-access memory (RRAM) offers additional advantages. For process and material versatility properties, RRAM can be fabricated from diverse functional materials, including metal oxides (HfO_2_, TiO_2_, SnO_2_, SrTiO_3_), sulfides, nitrides, and organics, many of which are CMOS-compatible. The simple device structure supports multiple deposition techniques such as sol–gel, sputtering, chemical vapor deposition (CVD), and atomic layer deposition (ALD). For electrical and operational flexibility properties, the precise control of conductive filament dimensions enables multi-level cell (MLC) storage, increasing density by storing multiple bits per cell. RRAM exhibits fast switching (nanoseconds–microseconds), supports both unipolar and bipolar modes, and offers non-destructive readout, preserving endurance. For energy efficiency and reliability, operating at low voltages with minimal power consumption, RRAM is suitable for low-power and IoT applications. It exhibits excellent data retention without power and endurance of up to 10^6^–10^9^ cycles, depending on material systems [5,6,7,8,9,10,11,12,13,14]. In addition, the previous study also found that related memory devices might be manufactured using electrochemical methods for generation and tuning of semiconductor electronic and functional properties through electrochemical patterning [15]. Its superior performance and scalability make it a compelling candidate for future mainstream memory applications, with strong potential to shape the next era of memory development.

## 2. Experiment Details

**Figure 1:**
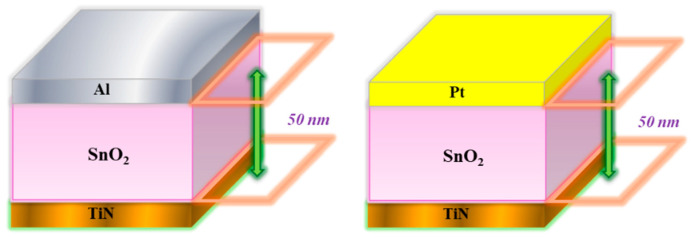
The diagram of Al/SnO_2_/TiN/SiO_2_/Si- and Pt/SnO_2_/TiN/SiO_2_/Si-structured RRAM devices.

In this study, tin oxide-based (SnO_2_) thin films were deposited as the insulating layer in metal–insulator–metal (MIM)-structured resistive random-access memory (RRAM) devices using radiofrequency (rf) magnetron sputtering. The SnO_2_ target was positioned at a distance of approximately 5 cm from the substrate to ensure uniform deposition. To eliminate intrinsic defects within the oxide target and to stabilize the plasma during the sputtering process, a pre-sputtering treatment was conducted for 20 min in a pure argon (Ar) atmosphere. The sputtering process utilized a 2-inch SnO_2_ ceramic target, with an applied rf power of 100 W, under a working gas mixture consisting of Ar and different oxygen concentrations. The deposition parameters were set to an rf power of 100 W, a chamber pressure of 20 mTorr, and a sputtering duration of 10 min. Deposition process was performed at room temperature. For the fabrication of the tin oxide-based resistive random-access memory (RRAM) structure, the tin oxide-based (SnO_2_) thin films were deposited on to TiN/SiO_2_/Si substrates using radiofrequency (rf) magnetron sputtering. Following the deposition process, the SnO_2_ films were subjected to rapid thermal annealing (RTA) to modify their physical and electrical properties. The post-deposition RTA process involved various annealing temperatures such as 500 °C, each with a treatment duration of 30 s, aimed at enhancing film crystallinity and reducing residual stress. For 30 s, the time of rapid thermal annealing process refers only to the dwell time at the target temperature, excluding heating and cooling. The heating rate was about 20 °C/s, followed by a 30 s hold, and natural cooling at 15 °C/s until <100 °C before removal. Subsequently, a circular-top electrode array (diameter = 0.1 cm) composed of aluminum and platinum was deposited using a metal shadow mask and dc sputtering under a pure argon (100% Ar) environment with an aluminum and platinum target. This process formed metal–insulator–metal (MIM) structures, denoted as Al/SnO_2_/TiN/SiO_2_/Si and Pt/SnO_2_/TiN/SiO_2_/Si structures, as illustrated in Figure 1.

To analyze the crystalline structure of the SnO_2_ thin films, X-ray diffraction (XRD) measurements were performed over a 2θ scan range of 20° to 60°. The surface morphology and cross-sectional structure of the films were examined using a scanning electron microscope (SEM). The current–voltage (*I*–*V*) switching behavior of the Al/SnO_2_/TiN/SiO_2_/Si- and Pt/SnO_2_/TiN/SiO_2_/Si-structured RRAM devices was characterized using Agilent B1500 semiconductor parameter analyzer to evaluate its resistive switching performance. The underlying carrier conduction mechanisms in both the set and reset states were thoroughly investigated and discussed. Finally, the optical transmittance of the fabricated Al/SnO_2_/TiN/SiO_2_/Si- and Pt/SnO_2_/TiN/SiO_2_/Si-structured RRAM structures was measured across the visible spectrum (400–800 nm) using a UV–VIS spectrophotometer, confirming its potential for transparent memory applications.

## 3. Results and Discussion

The crystalline structure of the tin oxide-based (SnO_2_) thin films was investigated using glancing-incidence X-ray diffraction (GIXRD). This technique was employed to enhance surface sensitivity and accurately characterize the crystallographic phases of the thin films. The measurements were conducted using an X-ray diffractometer operating at an accelerating voltage of 45 kV and a tube current of 120 mA. The X-ray source employed a copper anode (Cu Kα radiation, λ = 1.54184 Å). The scanning parameters were set with a step size of 0.05° and a dwell time of 3 s per step. The scanning range spanned from 20° to 80° (2θ), with a fixed glancing-incidence angle of 2° to ensure optimal surface diffraction signal detection.

**Figure 2:**
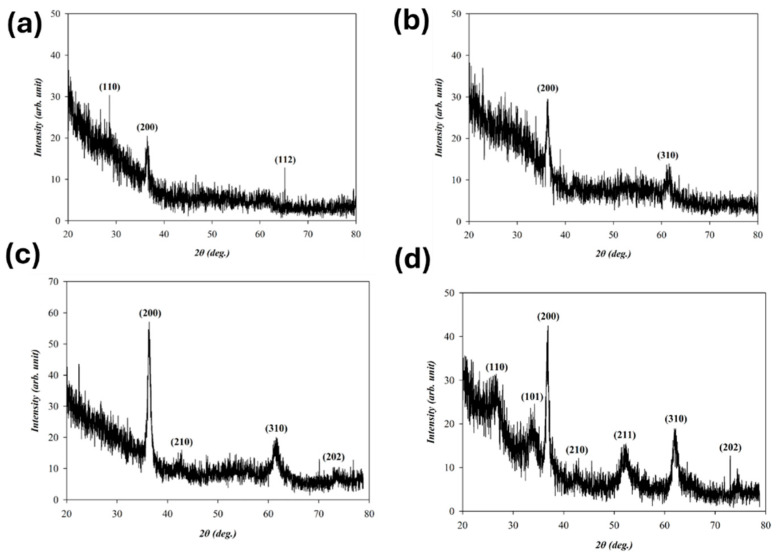
The XRD patterns of as-deposited tin oxide-based (SnO_2_) thin films for different oxygen-to-argon ratio gas composition deposition parameters: (**a**) (O_2_: 0 sccm; Ar: 20 sccm), (**b**) (O_2_: 0 sccm; Ar: 29 sccm), (**c**) (O_2_: 10 sccm; Ar: 10 sccm), and (**d**) RTA-treated conditions.

Figure 2a,b show the XRD patterns of SnO_2_ thin films deposited under oxygen concentration conditions with argon flow rates of 20 sccm and 29 sccm, respectively. Both tin oxide-based films exhibited polycrystalline structures. The 20 sccm sample showed random orientations with (110), (200), and (112) peaks, while the 29 sccm film exhibited a preferred (200) orientation along with (310), indicating improved texture. The latter also showed a narrower FWHM, a higher intensity, and a better crystallinity.

Figure 2a,c show the XRD patterns of SnO_2_ films deposited under 0% and 50% oxygen-to-argon ratios. Both films exhibited polycrystalline structures. The 0% oxygen film showed random orientations with (110), (200), and (112) peaks. In contrast, the 50% oxygen film displayed four peaks for (200), (210), (310), and (202), with a strong preferred (200) orientation. This was confirmed by a narrower FWHM and a higher intensity, indicating enhanced crystallinity due to improved oxidation during deposition.

Figure 2c,d show the XRD patterns of SnO_2_ thin films deposited with a 50% O_2_/Ar ratio before and after rapid thermal annealing (RTA). Both films exhibited polycrystalline structures. The as-deposited film showed four peaks for (200), (210), (310), and (202), with (200) as the preferred orientation. The RTA-treated film exhibited seven peaks, including (110), (101), and (211), indicating enhanced texturing. Although the (200) peak intensity slightly decreased after RTA, the narrower FWHM confirmed improved crystallinity, attributed to thermally induced atomic rearrangement and dislocation reduction.

**Figure 3:**
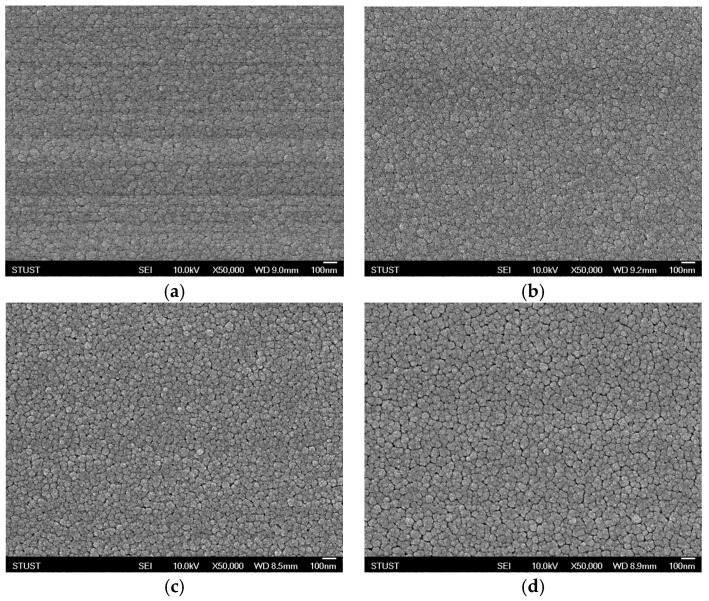
The FE-SEM images of as-deposited tin oxide-based (SnO_2_) thin films for different oxygen-to-argon ratio gas composition deposition parameters: (**a**) (O_2_: 0 sccm; Ar: 20 sccm), (**b**) (O_2_: 0 sccm; Ar: 29 sccm), (**c**) (O_2_: 10 sccm; Ar: 10 sccm), and (**d**) RTA-treated conditions.

**Figure 4:**
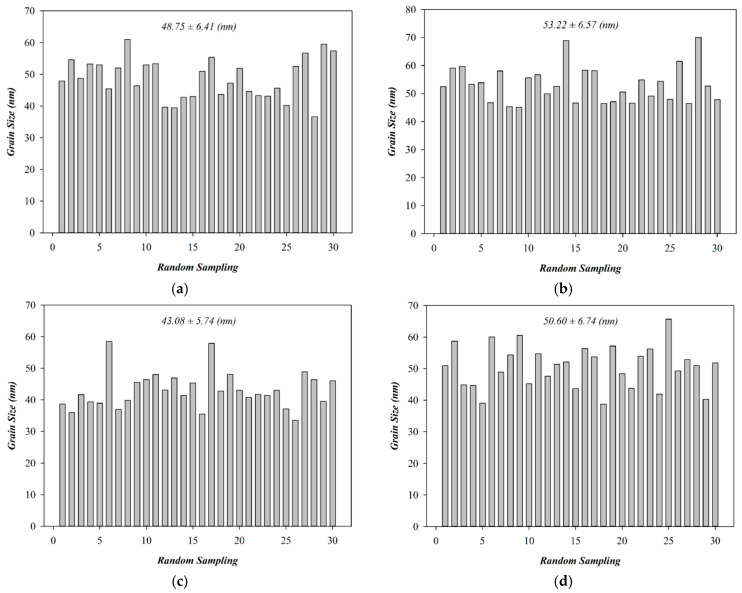
The mean standard deviation value of particle size of as-deposited tin oxide-based (SnO_2_) thin films for different oxygen-to-argon ratio gas composition deposition parameters: (**a**) (O_2_: 0 sccm; Ar: 20 sccm), (**b**) (O_2_: 0 sccm; Ar: 29 sccm), (**c**) (O_2_: 10 sccm; Ar: 10 sccm), and (**d**) RTA-treated conditions.

The surface morphology of the tin oxide-based (SnO_2_) thin films was analyzed using high-resolution field-emission scanning electron microscopy (HRFE-SEM) to obtain scanning electron images (SEIs) of the film surface. The imaging was performed under an accelerating voltage of 10.0 kV, with a magnification of ×50,000, and a working distance of 9.0 mm. These imaging conditions allowed for the detailed examination of the nanoscale surface features and microstructural characteristics of the SnO_2_ thin film surface. Figure 3a,b show the SEM images of SnO_2_ thin films deposited under oxygen concentration conditions with argon flow rates of 20 sccm and 29 sccm, respectively. Both films exhibited smooth, dense morphologies. The 20 sccm sample had agglomerated grains (48.75 ± 6.41 nm), while the 29 sccm film showed larger, more uniform grains (53.22 ± 6.57 nm) with clearer grain boundaries in Figure 4a,b. These results suggest that increasing argon flow promotes grain growth under oxygen concentration conditions. Figure 3a,c show the SEM morphologies of SnO_2_ thin films deposited with 0% and 50% O_2_/Ar flow ratios, respectively. Both films exhibited smooth, dense surfaces. The 0% oxygen film showed agglomerated grains (48.75 ± 6.41 nm), while the 50% oxygen film displayed smaller, uniform grains (43.08 ± 5.74 nm) with clearer boundaries in Figure 4c. The increased oxygen concentration reduced agglomeration by enhancing surface oxygen adsorption, promoting uniform grain formation and oxygen vacancies essential for resistive switching. The surface morphologies of SnO_2_ thin films with a 50% O_2_/Ar flow ratio, both as-deposited and post-annealed by rapid thermal annealing (RTA), were examined by SEM (Figure 3c,d). Both films showed smooth, dense surfaces, indicating good quality. The as-deposited film had uniform, rounded grains (43.08 ± 5.74 nm), while the RTA-treated film exhibited larger, agglomerated grains (50.60 ± 6.74 nm) with increased intergranular spacing in Figure 4c,d. The grain growth and agglomeration are attributed to thermal energy during annealing, which enhances lattice rearrangement and oxygen vacancy formation, beneficial for resistive switching conduction. Cooling of RTA process is critical for oxide crystallization, affecting grain boundaries, oxygen vacancies, and residual stress. For SnO_2_ films, no abnormal phase transitions were observed; however, consistent with prior reports, slower cooling improved crystallinity and reduced defects, while rapid cooling trapped vacancies, impacting switching stability. The chosen cooling profile optimized crystallinity and minimized defect formation, ensuring stable device performance [16,17,18].

In this study, for the set state, further progress pertaining to the different electrical conduction mechanisms will be defined and discussed from the *I*–*V* curves’ fitting of the different oxygen contents in SnO_2_-thin-film RRAM devices for using the RTA post-treatment and top electrode materials. The equations of ohmic, space-charge-limit-current, Schottky, and Poole–Frenkel transport mechanisms are described below.

The electrical current density of the ohmic conduction mechanism is written as Equation (1):

(1) $$J=E_{i}exp\left(\frac{-△E_{ac}}{kT}\right)$$

$E_{i}$ is the electric field in the insulator, $\;△E_{ac}$ is the electron activation energy, k is the Boltzmann constant, and T is the temperature [15].

The electrical current density of the space-charge-limit-current conduction mechanism is written as Equation (2):

(2) $$J_{SE}=A\times T^{2}exp\left(\frac{\beta_{SE}\sqrt{E}}{kT}\right)$$

$C_{t}$ is the trap density constant, E is the electric field in the insulator, q is the elementary charge of the carrier,$\;\Phi_{B}$ is the barrier height, ε is the dielectric constant of the insulator, $\;\varepsilon_{0}$ is the vacuum permittivity, k is the Boltzmann constant, and T is the temperature [15].

The electrical current density of the Schottky conduction mechanism is written as Equation (3):

(3) $$J_{SE}=A\times T^{2}exp\left(\frac{\beta_{SE}\sqrt{E}}{kT}\right)$$

$C_{RD}$ is the effective Richardson constant, q is the elementary charge of the carrier, m* is the effective mass of the carrier, k is the Boltzmann constant, h is the insulator thickness, $\Phi_{B}$ is the barrier height, T is the temperature, $\varepsilon_{0}$ is the vacuum permittivity, $\varepsilon_{r}\;$ is the dynamic dielectric constant (at a high frequency), and E is the electric field in the insulator [15].

The electrical current density of the Poole–Frenkel conduction mechanism is written as Equation (3):

(4) $$J=\frac{9\varepsilon_{i}uV^{2}}{8d^{3}}=\left(\frac{9\varepsilon_{i}u}{8d^{3}}\right)E_{i}^{2}$$

$\varepsilon_{i}$ is the dielectric constant, u is the carrier mobility, d is the insulator thickness, and $E_{i}$ and *V* are the electric field and voltage across the insulator [19].

**Figure 5:**
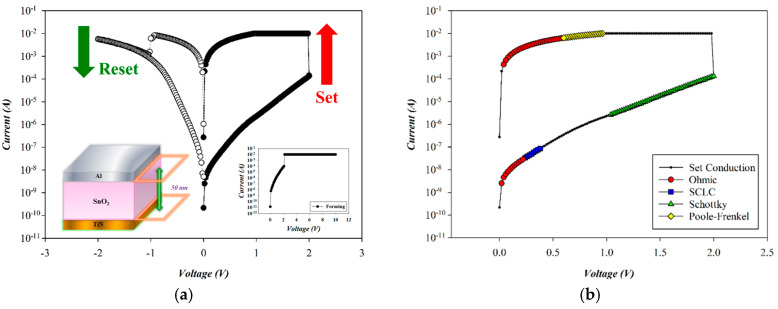
(**a**) The *I*–*V* switching curves and (**b**) electrical conduction mechanism of as-deposited tin oxide-based (SnO_2_) thin film (O_2_: 0 sccm; Ar: 20 sccm) RRAM devices for the set state.

Figure 5 illustrates the *I*–*V* switching behavior and conduction mechanism of as-deposited SnO_2_-based RRAM devices (O_2_: 0 sccm; Ar: 20 sccm) during the set process. A current compliance of 10 mA was applied to prevent hard breakdown. The forming process was triggered by a positive bias on the Al top electrode, resulting in soft breakdown at 2.1 V (inset of Figure 5). A full resistive switching cycle was achieved via a negative reset pulse followed by a positive set pulse. Bipolar voltage sweeps (±2 V) demonstrated stable switching, with reset and set voltages around −1 V and +2 V, respectively.

**Figure 6:**
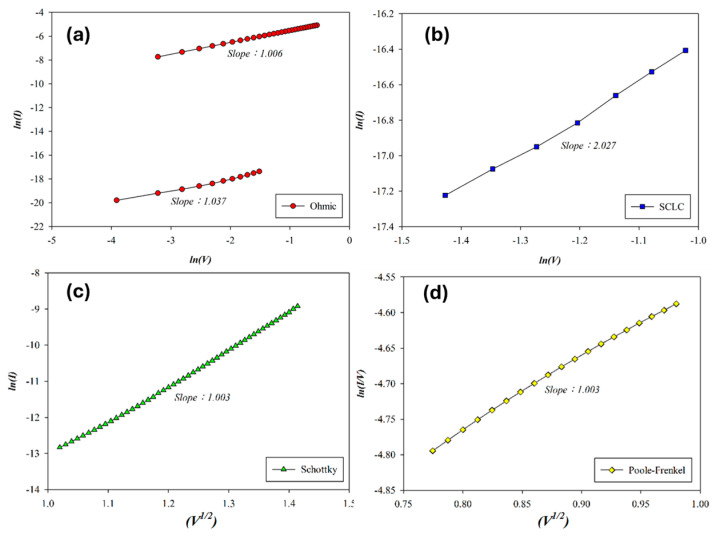
The *I*–*V* curves’ fitting of as-deposited tin oxide-based (SnO_2_) thin film (O_2_: 0 sccm; Ar: 20 sccm) RRAM devices for the set state for the (**a**) ohmic, (**b**) space-charge-limit-current, (**c**) Schottky, and (**d**) Poole–Frenkel conduction mechanisms.

The carrier transport mechanism of the Al-top-electrode SnO_2_ RRAM device (O_2_: 0 sccm; Ar: 20 sccm) during the set process was analyzed under forward bias, as shown in Figure 6. At low voltages, ohmic conduction dominates, while increased voltage and temperature induce a transition from Schottky to Poole–Frenkel emission at higher fields. In the high-resistance state (HRS), extracted slopes of 1.037, 2.027, and 1.003 correspond to ohmic conduction, space-charge-limited-current (SCLC), and Schottky emission in Figure 6a,b. In the low-resistance state (LRS), slopes of 1.003 and 1.006 indicate Poole–Frenkel and ohmic conduction in Figure 6a,d. According to the above *I*–*V* curves’ fitting, the SnO_2_ thin films (O_2_: 0 sccm; Ar: 20 sccm) exhibited excess oxygen vacancies from the Poole–Frenkel mechanism for the high voltage applied. We also speculated the ohmic mechanism attributed to and generated by oxygen ions at the interface of AlO_x_ from the Al-top-electrode/SnO_2_ films.

**Figure 7:**
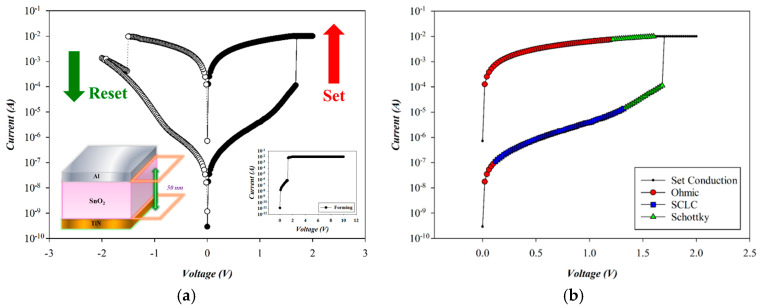
(**a**) The *I*–*V* switching curves and (**b**) electrical conduction mechanism of as-deposited tin oxide-based (SnO_2_) thin film (O_2_: 0 sccm; Ar: 29 sccm) RRAM devices for the set state.

Figure 7 shows the *I*–*V* switching characteristics and conduction mechanism of as-deposited SnO_2_-based RRAM devices (O_2_: 0 sccm; Ar: 29 sccm) during the set process. To prevent permanent breakdown, a current compliance of 10 mA was applied. A positive bias on the Al top electrode initiated the forming process, resulting in soft breakdown at ~1.9 V (in inset of Figure 7). Subsequent reset and set operations were performed via negative and positive biases, respectively, completing one switching cycle. Repeated bipolar sweeps between −2 V and +2 V confirmed stable switching, with reset and set voltages of approximately −1.5 V and +1.7 V.

**Figure 8:**
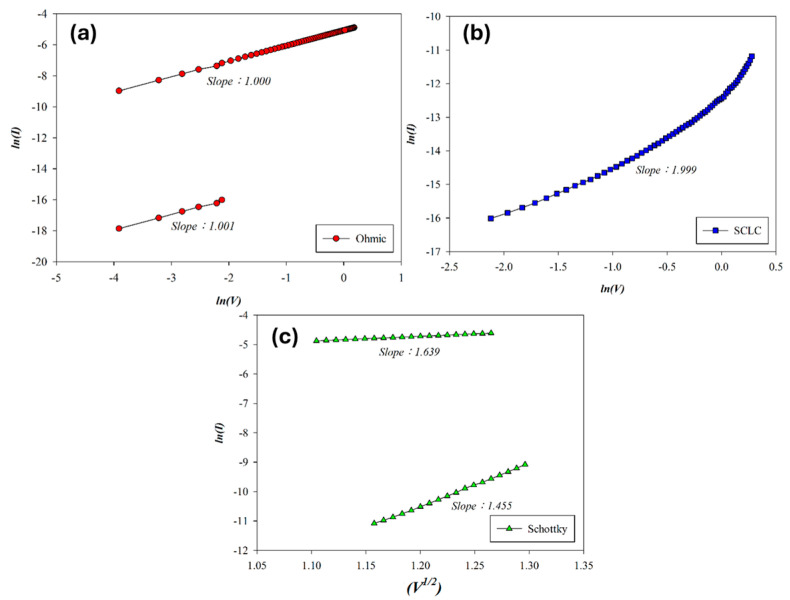
The *I*–*V* curves’ fitting of as-deposited tin oxide-based (SnO_2_) thin film (O_2_: 0 sccm; Ar: 29 sccm) RRAM devices for the set state for the (**a**) ohmic, (**b**) space-charge-limit-current, and (**c**) Schottky conduction mechanisms.

The carrier transport mechanisms during the set process of Al-top-electrode SnO_2_ RRAM (O_2_: 0 sccm; Ar: 29 sccm) were analyzed under a positive bias. As shown in Figure 8, ohmic conduction governs the low-voltage region, while elevated voltage induces temperature rise, transitioning the mechanism to Schottky emission. In the high-resistance state (HRS), slope values of 1.001, 1.999, and 1.455 indicate ohmic conduction, SCLC, and Schottky emission, respectively. In the low-resistance state (LRS), slopes of 1.639 and 1.000 correspond to Schottky emission and ohmic conduction, confirming voltage-dependent transport behavior. According to the above *I*–*V* curves’ fitting, the SnO_2_ thin films (O_2_: 0 sccm; Ar: 29 sccm) exhibited excess oxygen vacancies from the SCLC mechanism for the high voltage applied. We also speculated the ohmic mechanism attributed to and generated by oxygen ions at the interface of AlO_x_ from the Al-top-electrode/SnO_2_ films.

**Figure 9:**
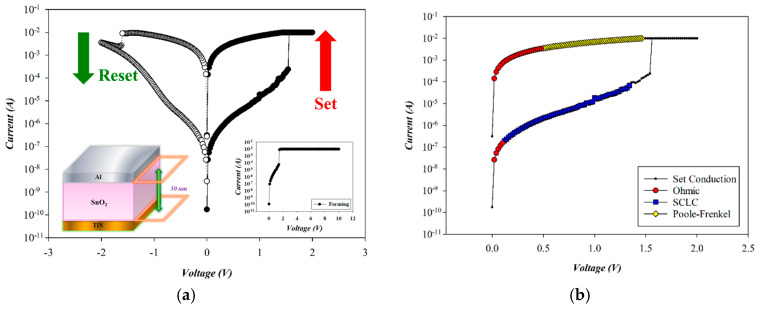
(**a**) The *I*–*V* switching curves and (**b**) electrical conduction mechanism of as-deposited tin oxide-based (SnO_2_) thin film (O_2_: 10 sccm; Ar: 10 sccm) RRAM devices for the set state.

Figure 9 illustrates the *I*–*V* switching characteristics and conduction mechanism of as-deposited SnO_2_-based RRAM devices (O_2_: 10 sccm; Ar: 10 sccm) in the set state. A current compliance of 10 mA was applied to prevent permanent breakdown during the forming process. Soft breakdown occurred at 1.6 V under a positive bias (the inset of Figure 9). The resistive switching cycle was completed via successive reset (−1.6 V) and set (+1.5 V) operations. Bipolar voltage sweeps between −2 V and +2 V confirmed stable switching behavior.

**Figure 10:**
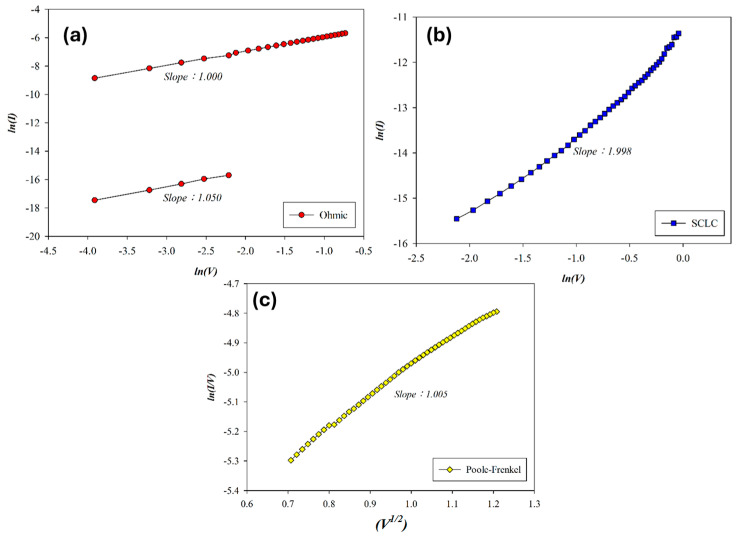
The *I*–*V* curves’ fitting of as-deposited tin oxide-based (SnO_2_) thin film (O_2_: 10 sccm; Ar: 10 sccm) RRAM devices for the set state for the (**a**) ohmic, (**b**) space-charge-limit-current, and (**c**) Poole–Frenkel conduction mechanisms.

The carrier transport mechanism of the Al/SnO_2_ RRAM device (O_2_: 10 sccm; Ar: 10 sccm) during the set process was analyzed under a positive bias. As shown in Figure 10a, ohmic conduction dominates at low voltages, transitioning to space-charge-limited-current (SCLC) and then Poole–Frenkel emission at higher fields due to an increased device temperature. In the high-resistance state (HRS), slopes of 1.050 and 1.998 correspond to ohmic and SCLC conduction. In the low-resistance state (LRS), slopes of 1.005 and 1.000 indicate Poole–Frenkel and ohmic behavior, respectively, as shown in Figure 10a,c. According to the above *I*–*V* curves’ fitting, the SnO_2_ thin films (O_2_: 10 sccm; Ar: 10 sccm) exhibited excess oxygen vacancies from the SCLC and Poole–Frenkel mechanisms for the high voltage applied.

**Figure 11:**
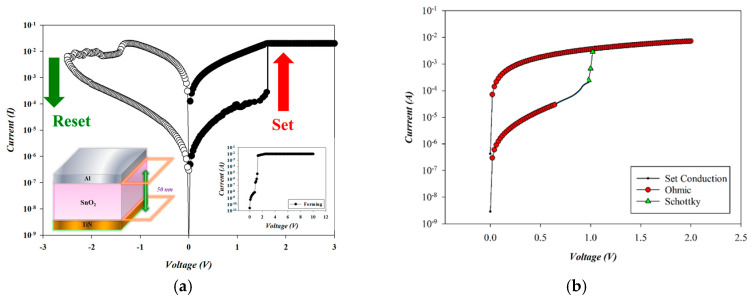
(**a**) The *I*–*V* switching curves and (**b**) electrical conduction mechanism of RTA-treated tin oxide-based (SnO_2_) thin film (O_2_: 10 sccm; Ar: 10 sccm) RRAM devices for the set state.

Figure 11 illustrates the *I*–*V* switching characteristics and conduction mechanism of RTA-treated SnO_2_-based RRAM devices (O_2_: 10 sccm; Ar: 10 sccm) in the set state. A current compliance of 10 mA was applied to prevent permanent breakdown during the forming process. A positive bias on the Al top electrode initiated soft breakdown at 2.4 V, as in the inset of Figure 11a. The reset and set operations were then completed by applying successive negative and positive biases, forming a full switching cycle. Subsequent bipolar voltage sweeps (−2 V to +2 V) confirmed stable resistive switching behavior, with typical reset and set voltages of −1.4 V and +1.4 V, respectively.

**Figure 12:**
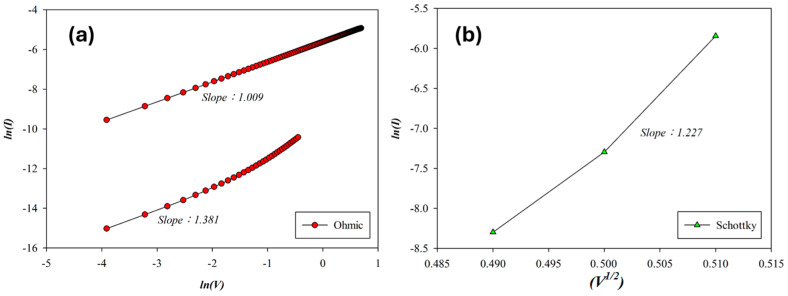
The *I*–*V* curves’ fitting of RTA-treated tin oxide-based (SnO_2_) thin film (O_2_: 10 sccm; Ar: 10 sccm) RRAM devices for the set state for the (**a**) ohmic and (**b**) Schottky conduction mechanisms.

The carrier transport mechanism during the set process of the RTA-treated SnO_2_-thin-film device (O_2_: 10 sccm; Ar: 10 sccm) with an Al top electrode was analyzed under a positive bias. As shown in Figure 12a, ohmic conduction dominated both low and high voltage regions, while Schottky emission appeared during the intermediate regime due to temperature rise. In the high-resistance state (HRS), slope values of 1.381 and 1.227 corresponded to ohmic conduction and Schottky emission, respectively. In the low-resistance state (LRS), a slope of 1.009 confirmed ohmic behavior. According to the above *I*–*V* curves’ fitting, the oxygen vacancies and defects of RTA-treated SnO_2_ thin films (O_2_: 10 sccm; Ar: 10 sccm) decreased by the RTA post-treatment process due to the ohmic conduction mechanism.

**Figure 13:**
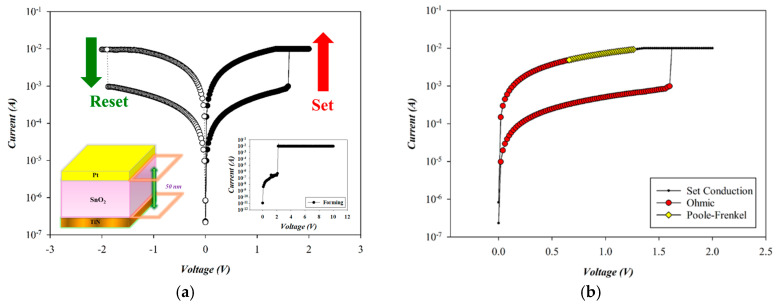
(**a**) The *I*–*V* switching curves and (**b**) electrical conduction mechanism of tin oxide-based (SnO_2_) thin film (O_2_: 10 sccm; Ar: 10 sccm) RRAM devices using a platinum electrode for the set state.

Figure 13 presents the *I*–*V* switching characteristics and conduction mechanism of RTA-treated SnO_2_-based RRAM devices (O_2_: 10 sccm; Ar: 10 sccm) in the set state. To prevent permanent breakdown from sudden current surges, a current compliance of 10 mA was applied. A positive bias was then applied to the Pt top electrode, inducing soft breakdown at 2.1 V, as in the inset of Figure 13. A negative bias was used for the reset operation, followed by a positive bias for the set process, completing a full reset/set cycle. Subsequent bipolar voltage sweeps from −2 V to +2 V revealed typical reset and set voltages of approximately −1.8 V and +1.6 V, respectively.

**Figure 14:**
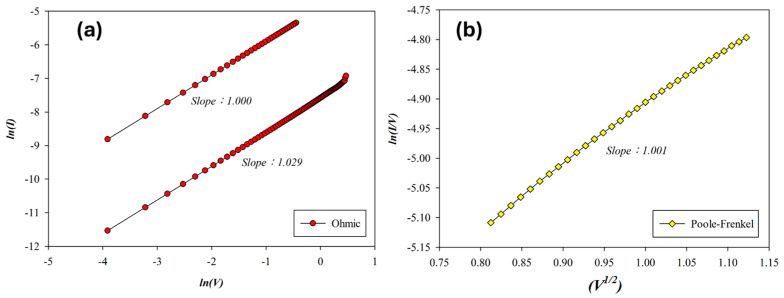
The *I*–*V* curves’ fitting of tin oxide-based (SnO_2_) thin film (O_2_: 10 sccm; Ar: 10 sccm) RRAM devices using a platinum electrode for the set state for the (**a**) ohmic and (**b**) Poole–Frenkel conduction mechanisms.

The carrier transport mechanism during the set process of the Pt-top-electrode SnO_2_ thin-film device (O_2_: 10 sccm; Ar: 10 sccm) was examined under a positive bias. As shown in Figure 14a, ohmic conduction dominates at low voltages. With increasing voltage and temperature, conduction transitions to Poole–Frenkel emission at higher fields in Figure 14b. In the high-resistance state (HRS), a slope of 1.029 indicates ohmic conduction. In the low-resistance state (LRS), extracted slopes of 1.001 and 1.000 correspond to Poole–Frenkel emission and ohmic conduction, respectively. According to the above *I*–*V* curves’ fitting, the oxygen vacancies and defects of RTA-treated SnO_2_ thin films (O_2_: 10 sccm; Ar: 10 sccm) decreased by the RTA post-treatment process due to the ohmic conduction mechanism.

**Figure 15:**
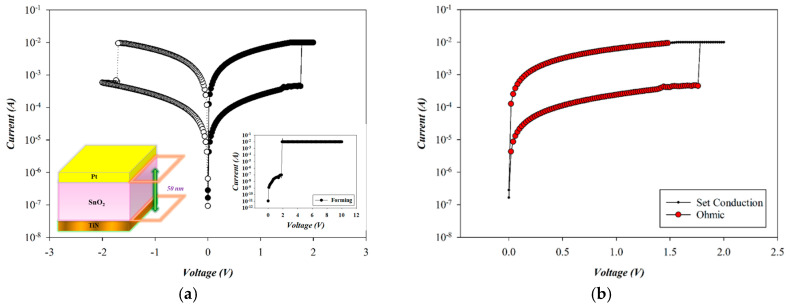
(**a**) The *I*–*V* switching curves and (**b**) electrical conduction mechanism of RTA-treated tin oxide-based (SnO_2_) thin film (O_2_: 10 sccm; Ar: 10 sccm) RRAM devices using a platinum electrode for the set state.

Figure 15 shows the *I*–*V* switching characteristics and conduction mechanism of RTA-treated SnO_2_-based RRAM devices (O_2_: 10 sccm; Ar: 10 sccm) using a platinum electrode in the set state. To prevent permanent breakdown from a sudden current surge, a current compliance of 10 mA was applied. A positive bias was then applied to the Pt top electrode to initiate the forming process, resulting in soft breakdown at approximately 1.8 V, as in the inset of Figure 15. Subsequent reset and set operations were carried out by applying negative and positive biases, respectively, completing one full resistive switching cycle. The device was then subjected to repeated bipolar voltage sweeps from −2 V to +2 V for *I*–*V* characterization. Typical reset and set voltages were approximately −1.7 V and +1.8 V, respectively.

**Figure 16:**
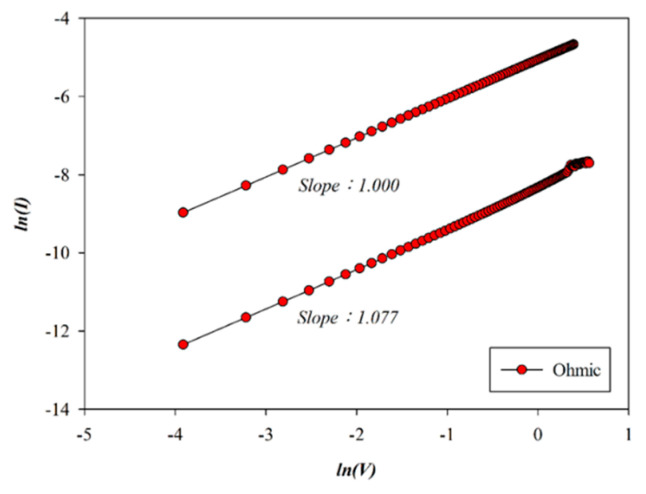
The *I*–*V* curves’ fitting of RTA-treated tin oxide-based (SnO_2_) thin film (O_2_: 10 sccm; Ar: 10 sccm) RRAM devices using a platinum electrode for the set state for the ohmic conduction mechanism.

The carrier transport mechanism during the set process of the RTA-treated Pt-top-electrode SnO_2_-thin-film device (O_2_: 10 sccm; Ar: 10 sccm) was investigated under a positive bias. As shown in Figure 16, ohmic conduction dominated across both low- and high-voltage regions. In the high-resistance state (HRS), a slope of 1.077 was observed, while in the low-resistance state (LRS), the slope was 1.000, both confirming ohmic behavior, as illustrated in Figure 16. Similarly, oxygen vacancies and defects of RTA-treated SnO_2_-thin-film RRAM devices also decreased by the RTA post-treatment process due to the ohmic conduction mechanism by using a platinum top electrode. We speculated that the symmetry bipolar switching curve properties of RRAM devices were attributed to using a platinum top electrode and not to the AlOx interface effect. The relevant proof will be confirmed later in the electrical filament of the physical model.

**Figure 17:**
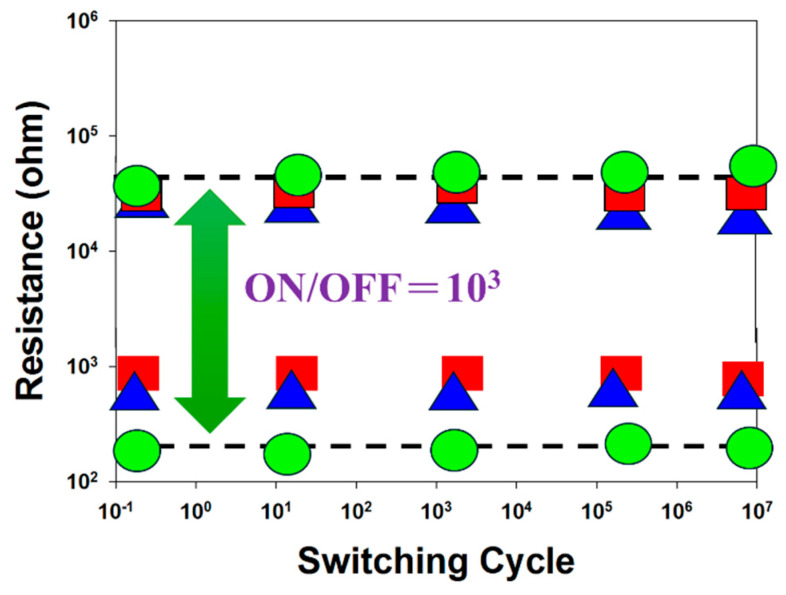
The resistance value versus switching cycle curves of the tin oxide-based (SnO_2_) thin film RRAM devices (red: 10% oxygen content; blue: an RTA-treated film; green: a platinum electrode).

**Figure 18:**
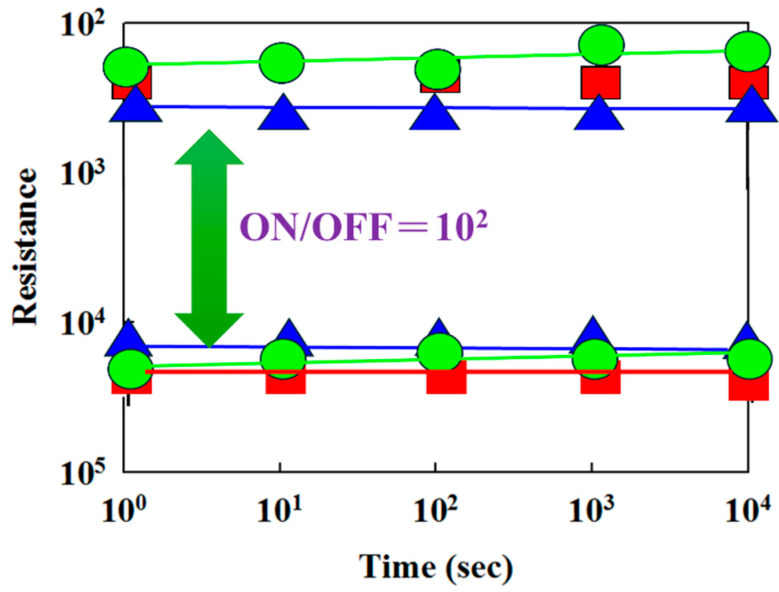
The resistance value versus time curves of tin oxide-based (SnO_2_) thin film RRAM devices (red: 10% oxygen content; blue: an RTA-treated film; green: a platinum electrode).

Figure 17 presents the resistance versus switching cycle characteristics of tin oxide-based RRAM devices for different deposition parameters and top electrode materials (such as Al and Pt), where red, blue, and green curves represent 10% oxygen content, an RTA-treated film, and a platinum electrode, respectively. The extrapolated data indicate stable on/off switching behavior over a period exceeding 10^3^ s. Figure 18 illustrates the resistance retention and endurance performance of the tin oxide-based RRAM devices, evaluated through repeated switching cycles. The retention characteristics for different deposited parameters and top electrode materials were assessed to determine their reliability for non-volatile memory applications. According to the extrapolated results, both devices maintained stable on/off resistance ratios without significant degradation for durations exceeding 10^2^ s.

**Figure 19:**
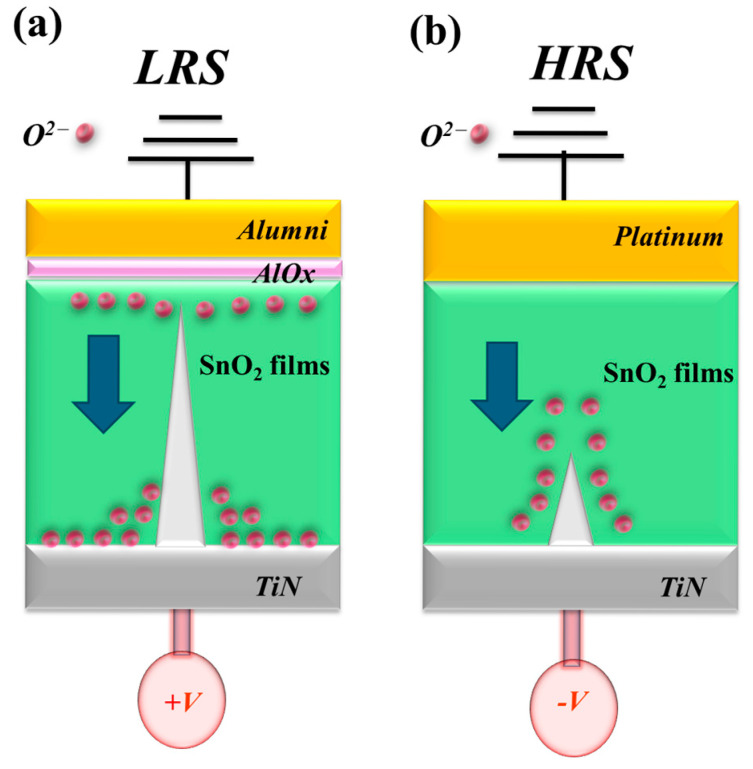
The initial metallic filament path model of the transparent ITO/ITOX/ITO RRAM device for the (**a**) LRS (set) and (**b**) HRS (reset) state.

Figure 19 illustrates the formation and evolution of oxygen vacancies at the interface between the aluminum (Al) and platinum (Pt) top electrodes and the tin oxide thin film in the RRAM device, particularly during the low-resistance state (LRS), in which these vacancies progressively accumulate. Under the influence of a high positive bias applied to the bottom electrode, a continuous oxidation reaction occurs along the conductive metallic filament, modulating the device’s resistive switching behavior. The presence of oxygen atoms near the bottom electrode region affects the growth and stability of these filaments. The schematic in Figure 19 depicts the initial formation and conduction pathway of metallic filaments, along with the corresponding charge transport mechanisms in Al(Pt)/BST/TiN-based RRAM devices during the set processes. For Al-top-electrode configurations, all Pt-based devices primarily exhibit ohmic conduction at a low bias, which is attributed to the presence of interfacial defects and elevated leakage currents at the top electrode and the tin oxide interface.

According to the summary in Table 1, electrical performance, including *I*–*V* characteristics, endurance, switching ratio, and conduction mechanisms, was evaluated using a semiconductor parameter analyzer [19,20]. In Table 1, all devices showed on/off ratios around 10^2^, with set/reset voltages below 2 V. Low-voltage conduction was dominated by ohmic or space-charge-limited-current (SCLC) conduction mechanisms. High-voltage conduction was mainly governed by Schottky or Poole–Frenkel emission. In addition, the RTA treatments promoted surface recrystallization and lattice defect repair, often resulting in ohmic conduction behavior.

For SnO_2_-based RRAM devices, optimal performance was achieved with an Al/SnO_2_/TiN stack fabricated under a moderate oxygen concentration (1:1 O_2_/Ar ratio) and short-duration rapid thermal annealing, enabling low forming voltage (2 V), stable bipolar switching, and on/off ratios of 10^2^. The simple MIM structure benefits from the Al electrode’s oxygen-gettering and the TiN electrode’s oxygen-reservoir properties, while avoiding excessive annealing that causes grain coalescence and cracks. Device stability was further enhanced by controlling compliance current during the forming process/the set state to prevent hard breakdown, targeting operation below 2 V. Optional interface layers (Ti, Al) can further lower *V*set and improve switching uniformity, while a diffusion barrier was essential for Cu-based electrodes.

## 4. Conclusions

In this study, silicon wafers were used as substrates, on which SiO_2_ and TiN thin films were deposited as the insulating layer and bottom electrode, respectively. Tin oxide (SnO_2_) films were then deposited using rf sputtering under three different process conditions to serve as the resistive switching layer. Subsequently, the tin oxide-based films underwent post-treatment to repair defects and rearrange the crystallization structure. Al and Pt top electrodes were formed via thermal evaporation and sputtering through a metal shadow mask, respectively, completing the metal–insulator–metal (MIM)-structured RRAM devices. In addition, the resistive switching characteristics of the tin oxide-based memory structures of RRAM devices using aluminum- and platinum-top-electrode materials were analyzed. The effects of different oxygen concentration deposited parameters and RTA process on the tin oxide-based films were investigated, and the memory characteristics under the two different top-electrode materials were compared.

The symmetry and non-symmetry bipolar switching *I*–*V* curves’ characteristics, the electrical conduction mechanism, and the different deposited oxygen concentrations of the tin oxide-based memory devices using rapid thermal annealing and different top electrodes were determined and investigated by ohmic, space-charge-limit-current, Schottky, and Poole–Frenkel conduction equations. From the *I*–*V* curves’ fitting, the low-voltage conduction of the RRAM devices was dominated by the ohmic/space-charge-limited-current (SCLC) mechanism. High-voltage conduction was mainly governed by Schottky/Poole–Frenkel emission. In addition, the RTA treatments promoted surface recrystallization and lattice defect repair, often resulting in ohmic conduction behavior in the set state of the RRAM devices. Based on electrical measurements of resistive switching behavior, the Al/SnO_2_/TiN-structured RRAM devices demonstrated a reliable operation exceeding 10^6^ cycles at different operation switching cycle times, with optimal performance conditions. At this condition, the devices exhibited approximately 10^2^ switching cycles, an on/off ratio of 2, and an operating voltage of around 2 V. Similarly, the Pt/SnO_2_/TiN RRAM devices reached up to 10^2^ cycles, with a high on/off ratio of 3 and a high operating voltage of 2 V. Moreover, the observed bipolar switching behavior in the tin oxide-based films confirms their functionality, supporting their use not only in resistive memory but also in micro-electronic applications. Given these favorable properties, the tin oxide-based RRAM devices show strong potential for future integration in CMOS-compatible systems and as candidates for next-generation resistance memory architectures.

## Figures and Tables

**Table 1:** The comparison of the electrical parameters and conduction mechanisms of tin oxide-based (SnO_2_) thin film RRAM devices.

	V_set_	V_reset_	O_n_/O_ff_ Ratio	I_on_	I_off_	Electrical Conduction Mechanism
O_2_: 0; Ar: 20 sccm	2 V	−1 V	10^2^	10^−2^	10^−4^	LRS: ohmic/Poole–Frenkel
						HRS: ohmic/Schottky
O_2_: 0; Ar: 29 sccm	1.7 V	−1.5 V	10^2^	10^−2^	10^−4^	LRS: ohmic/Schottky
						HRS: SCLC/Schottky
O_2_: 10; Ar: 10 sccm	1.5 V	−1.6 V	10^2^	10^−2^	10^−4^	LRS: ohmic/Poole–Frenkel
						HRS: SCLC
RTA conditions	1.4 V	−1.4 V	10^2^	10^−2^	10^−4^	LRS: ohmic
						HRS: ohmic
Using platinum electrode	1.6 V	−1.8 V	10^2^	10^−2^	10^−4^	LRS: ohmic/Poole–Frenkel
						HRS: ohmic
Using platinum electrode for RTA	1.8 V	−1.7 V	10^2^	10^−2^	10^−4^	LRS: ohmic
						HRS: ohmic

## Data Availability

Data is contained within the article.
